# Atomistic Investigation of the Titanium Carbide MXenes under Impact Loading

**DOI:** 10.3390/nano12142456

**Published:** 2022-07-18

**Authors:** Kang Xia, Haifei Zhan, Xinjie Zhang, Zhiyong Li

**Affiliations:** 1 College of Mechanical & Electrical Engineering, HoHai University, Nanjing 210098, China; xj.zhang@hhu.edu.cn; 2 School of Mechanical, Medical and Process Engineering, Queensland University of Technology (QUT), Brisbane, QLD 4001, Australia; zhiyong.li@qut.edu.au; 3 College of Civil Engineering and Architecture, Zhejiang University, Hangzhou 310058, China

**Keywords:** titanium carbide MXene, hypervelocity impact, molecular dynamics simulation

## Abstract

2D Titanium carbide MXenes with a structural formula recognized as Ti_n+1_C_n_ have attracted attention from both the academic and industry fields due to their intriguing mechanical properties and appealing potential in a variety of areas such as nano-electronic circuits/devices, bio sensors, energy storage and reinforcing material for composites. Based on mutli-body comb3 (third-generation Charge-Optimized Many-Body) potential, this work investigated the impact resistance of monolayer Ti_n+1_C_n_ nanosheets (namely, Ti_2_C Ti_3_C_2_ and Ti_4_C_3_) under hypervelocity up to 7 km/s. The deformation behavior and the impact resist mechanisms of Ti_n+1_C_n_ nanosheets were assessed. Penetration energy is found to positively correlate with the number of titanium atom layer (*n*). However, in tracking atomic Von Mises stress distribution, Ti_2_C exhibits the most significant elastic wave propagation velocity among the examined nanosheets, suggesting the highest energy delocalization rate and stronger energy dissipation via deformation prior to bond break. Consistently, Ti_2_C presents superior specific penetration energy due its Young’s-modulus-to-density ratio, followed by Ti_3_C_2_ and Ti_4_C_3_, suggesting an inverse correlation between the titanium atom layer number and specific penetration energy. This study provides a fundamental understanding of the deformation and penetration mechanisms of titanium carbide MXene nanosheets under impact, which could be beneficial to facilitating their emerging impact protection applications.

## 1. Introduction

The first phenomenal 2D material, graphene, is renowned for its record-breaking properties [1]. Followed by its discovery, tremendous efforts on the exploration of graphene-analogous 2D carbonaceous materials and transition metal carbides/nitrides (widely recognized as MXenes) are conducted. MXenes are 2D-layered transition metal carbon/nitride materials first realized by selectively washing out element ‘A’ in the atomically layered MAX phase with the etching approach [2], where ‘M’, ‘A’, ‘X’ represent an early transition metal, an A-group metal element (more specifically, a group IIIA or IVA element) and a C and/or N element, respectively [3]. As a classical 2D nanomaterial, a strong binding energy from the M-X valence bond leads to strong mechanical properties in MXene [4].

Titanium carbide MXenes can be represented as Ti_n+1_C_n_ and have received immense attention from various fields, including the civil, automobile, aerospace and military industries, owing to their superior bending rigidity [5], Young’s modulus, strength-to-weight ratio [6], oil/water separation capability [7], electro-chemical performance [8], etc. To date, Ti_n+1_C_n_ has been realized via either top-down or bottom-up techniques. Representative top-down synthesis approaches include fluorine-based selective etching, which usually induces oxygen-containing termination functional groups. In contrast, bottom-up synthesis methods such as chemical vapor deposition can produce ‘pristine’ Ti_n+1_C_n_, with its surface not terminated. Considering the product quality and experiment setup difficulty, the top-down approach is clearly the main stream [9,10].

Varieties of Ti_n+1_C_n_ have been successfully realized so far; however, constrained by the lateral size of sheets and the complex oxygen-containing function groups observed in the synthesis process, most studies on monolithic Ti_n+1_C_n_ are carried out via the in silico approach [10]. Deploying the empirical potential energy function and embedded atom method, the molecular dynamics (MD) study suggests that the bending rigidity of these 2D titanium carbides can be as high as ~49.55 eV, which is significantly larger than that of other popular 2D materials such as graphene and MoS_2_ (whose bending rigidities are calculated to be 2.3 and 9.61 eV, respectively) [5]. A recent MD study also revealed that the Young’s modulus of Ti_n+1_C_n_ ranges between 133 and 517 GPa, which indicates its potential usages in nano-electronics and energy storage [6]. In addition to the research on pristine Ti_n+1_C_n_, a density functional theory (DFT) study on functionalized Ti_n+1_C_n_ suggested that the oxygen group not only results in strong anisotropy but also enhances their ideal tensile strength [1].

Besides its outstanding mechanical properties, Ti_n+1_C_n_ is also renowned as a strengthening material/matrix in a variety of composites and facilitates applications in nanogenerators, EMI shielding and sensors. The negatively charged MXene, due to the presence of oxygen-containing functional groups, is able to form a strong interaction with positively charged polymers, which improves the interfacial strength and interfacial load transfer efficiency [11]. In fact, lamellar composites with a stronger interfacial strength possess superior mechanical properties when subjected to quasi-static loading conditions [12].

Considering the outstanding mechanical behavior of Ti_n+1_C_n_ under the quasi-static loading condition, its performance under impact loading is rarely discussed in the literature; however, it is critical to facilitate its application in mechanical energy storge and bullet proof-related applications [12,13,14]. Due to the experimental complexity and small dimensions of available MXenes sheets [12], a pilot study on their impact resistance under various impact velocities is carried out utilizing the MD method in this work, with an emphasis on its deformation process, stress distribution capability and specific penetration energy.

## 2. Materials and Methods

**Figure 1:**
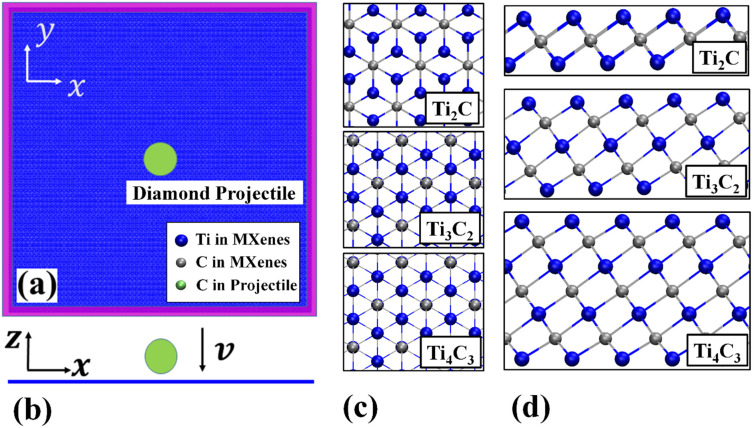
Impact simulation setup for Mxenes. (**a**) Top view of the sample, the magenta area indicates the fixed boundary and the green object is the diamond projectile; (**b**) Side view of the sample; (**c**,**d**) show the top and front view of the atomic structures for Ti_2_C Ti_3_C_2_ and Ti_4_C_3_, respectively.

The anti-ballistic performance and fracture behavior of Ti_n+1_C_n_ nanosheets subject to a hypervelocity impact are examined through MD simulations utilizing the open-source package LAMMPS [15]. Diamond is considered one of the strongest materials on earth and can stand hypervelocity impacts without cracking [16,17,18]; thus, a projectile with a diamond lattice structure is prepared for the impacts. The projectile is made up of 11,543 carbon atoms with a spherical shape of approx. 25 Å in radius. Square Ti_n+1_C_n_ nanosheets with fixed boundaries (highlighted with magenta in Figure 1) have identical planar dimensions of 500 × 500$\;{Å}^{2}$(containing 95,616, 159,360 and 214,508 atoms, respectively, for *n* = 1 to 3) [19]. High initial velocities up to 70 Å/ps (i.e., 7 km/s) are assigned to the projectile, with its initial bottom positions ~18 ${Å}^{2}$ above the geometric center of the nanosheet. The purpose of assigning an initial velocity to a free-standing projectile is to trace its energy variation during the whole impact process [18,20,21]. For comparison, projectile energy tracing is not achievable in LAMMPS utilizing a pure force, high speed indentation setup.

In this study, the third-generation Charge-Optimized Many-Body (COMB3) potential is employed to describe the C and Ti atomic interactions within the MXene nanosheets, as it is fully optimized to determine the binding energy of carbon- and metal-based systems [22,23,24,25,26]. The general form of COMB3 potential is defined as follows:

(1) $$\begin{array}{c} E_{COMB3}=\underset{i}{\sum }[E_{i}^{self}\left(q_{i}\right)+\underset{j>i}{\sum }\left[E_{ij}^{short}\left(r_{ij},q_{i},q_{j}\right)+E^{Coul}\left(r_{ij},q_{i},q_{j}\right)\right]+E^{polar}\left(q_{i},r_{ij}\right)+\\ \;\;\;\;\;\;\;\;\;\;\;\;\;\;\;\;\;\;\;\;\;\;\;E^{vdW}\left(\left\{r_{ij}\right\}\right)+E^{barr}\left(\left\{r_{ij}\right\}\right)+E^{corr}\left(\left\{r_{ij},\theta_{ijk}\right\}\right)] \end{array}$$

where $E^{self}$ is an electrostatic term of atom *i*, which is the sum of the atomic ionization energies and electron affinities. $E^{SHORT}$ presents the bond-order potential between the atom *i* and *j*. $E^{Coul},E^{vdW}$, $E^{barr}$ and $E^{CORR}$ are the Coulomb, van der Waals, charge barrier function and correction energy term, respectively. $E^{polar}$ is a term describing the polarization in an organic system which is excluded in this study. A modified Tersoff potential with a cutoff distance extended to 2.45 Å has been proven to represent the binding energy for diamond structures well [27], and it is adopted to mimic the atomic force within the diamond projectile. As the diamond projectile approaches MXene nanosheets, the ‘weak’ van der Walls force/interactions between the two structures are described by a Morse potential, which has been successfully used for diamond-involved contact simulation such as indentation [28,29], collision [30] and machining [31]. The general form of Morse potential is expressed as:

(2) $$V\left(r_{ij}\right)=D\left(e^{-2\alpha \left(r_{ij}-r_{0}\right)}-2e^{-\alpha \left(r_{ij}-r_{0}\right)}\right)$$

where α, D and $r_{0}$ represent range parameter, dissociation energy and equilibrium internuclear distance, respectively. The parameters in the Morse potential used for C–C interaction are: α = 2.624 ${Å}^{-1}$, D = 0.650 eV and $r_{0}$ = 2.000 Å [32]. Considering the C–Ti interaction, these three parameters are 1.900 ${Å}^{-1}$, 0.0137 eV and 2.867 Å, respectively [31].

During the relaxation stage, by employing non-periodic boundary conditions, an NVT (canonical) ensemble, a conjugate gradient algorithm and a Nose–Hoover thermostat [33], the whole system is equilibrated for 4000 fs at a low environment temperature of 10 K to achieve a minimum energy state. For the impact stage, a non-periodic boundary and a small time step of 0.1 fs are selected for the simulation in order to capture the dramatic deformation of the nanosheets. A time step of 0.5 fs is also employed to validate the setup, and similar results are obtained. The equations of motion are integrated with time using a velocity Verlet algorithm [34]. Temperature has great impacts on material strength, and they are negatively correlated for most materials [35,36]. For a fair comparison, the environment temperature is set to be a low value of 10 K for all the cases (20 K and 30 K are also tested, with similar simulation results obtained), aiming to minizine the influence of high thermal fluctuations (generated via impact), which may potentially weaken the material strength. To mimic the energy conversion between the kinetic and potential energy, the NVE (microcanonical) ensemble is chosen, and the thermostat is not applied to the system, during the entire impact stage.

The atomic stress in this work is calculated according to the virial stress $\Pi^{\alpha \beta }$, which is expressed as [37]:

(3) $$\Pi^{\alpha \beta }=\frac{1}{\Omega }\left(-\underset{i}{\sum }m_{i}v_{i}^{\alpha }v_{i}^{\beta }+\frac{1}{2}\underset{i}{\sum }\underset{j\neq i}{\sum }F_{ij}^{\alpha }r_{ij}^{\beta }\right)$$

Here, Ω donates the volume of the structure. $m_{i}$ and $v_{i}$ are the mass and velocity of the *i^th^* atom, respectively. $F_{ij}$ and $r_{ij}$ are the force and distance between atoms *i* and *j*, respectively, and the indices α and β denote the Cartesian components. The volume of the 2D Ti_n+1_C_n_ nanosheets is estimated by assuming them to be continuum media with different thicknesses. Adopting a different volume alters the stress calculation; however, the Ti_n+1_C_n_ nanosheets adopted in the work share similar lattice structures and densities. For the reason above, the volume differences will not change the trends of the results presented in this paper. Considering the complicated stress variation during impacts, Von Mises stress $\sigma_{VM}$ in the Ti_n+1_C_n_ is traced based on the atomic virial stress tensor. The tensor for each atom used for $\sigma_{VM}$ computation is a six-element vector, including three normal stresses—$\sigma_{x}$, $\sigma_{y}$, $\sigma_{z}$—and three planar stresses—$\sigma_{xy}$, $\sigma_{xz}$, $\sigma_{yz}$. The $\sigma_{VM}$ is calculated from:

(4) $$\sigma_{VM}=\sqrt{({\left(\sigma_{x}-\sigma_{y}\right)}^{2}+{\left(\sigma_{y}-\sigma_{z}\right)}^{2}+{\left(\sigma_{z}-\sigma_{x}\right)}^{2}+6(\sigma_{xy}{}^{2}+\sigma_{xz}{}^{2}+\sigma_{yz}{}^{2}))/2}$$

**Figure 2:**
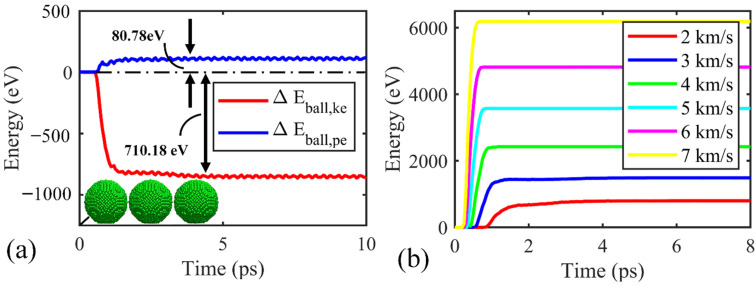
Energy variation over time for the projectile impact with the Ti_2_C membrane. (**a**) Energy change in the projectile as a function of time for an impact velocity of 2 km/s. ∆*E_ball,ke_* and ∆*E_ball,pe_* represent the kinetic and potential energy change of the projectile, respectively. (**b**) Dissipated energy in the projectile (∆*E_ball,tot_*) during the impact process.

Initially, the impact performance of the Ti_2_C nanosheet under an impact velocity of 2 km/s is highlighted. In the vacuum environment, the total energy change in the projectile equals the energy change in the Ti_2_C nanosheet. During the impact process, although the projectile experiences ignorable deformation, a notable amount of potential energy change of about 80.78 eV in the projectile (∆*E_ball,pe_*) is observed (Figure 2a). After perforation, the total energy loss in the projectile (∆*E_ball,tot_*), which is the sum of ∆*E_ball,pe_* and ∆*E_ball,ke_*, remains constant (Figure 2b). Thus, ∆*E_ball,tot_* is taken as the penetration energy (*E_p_*) rather than ∆*E_ball,ke_* alone [16,17].

## 3. Results and Discussion

### 3.1. Deformation Characteristics

**Figure 3:**
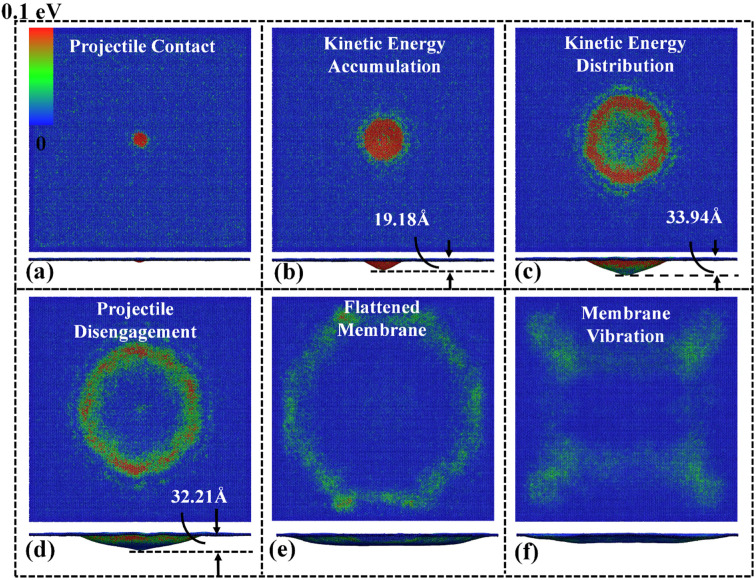
Impact deformation and kinetic energy distribution of the Ti_2_C nanosheet under an impact velocity of 0.4 km/s. Upper panels and bottom panels are the top and side views of the nanosheets, respectively. The kinetic energy distribution at the simulation times of: (**a**) 7.5 ps; (**b**) 12.5 ps; (**c**) 22.5 ps; (**d**) 37.5 ps; (**e**) 62.5 ps; and (**f**) 102.5.

To acquire the deformation process, the atomic configurations of three Ti_n+1_C_n_ MXenes (Ti_2_C, Ti_3_C_2_, Ti_4_C_3_) under impact velocities ranging from 0.1 km/s up to 7 km/s are investigated. Figure 3 illustrates the deformation of the Ti_2_C nanosheet subjected to a low impact velocity of ~0.4 km/s, and no bond break is observed during the whole impact process. Six deformation stages can be identified, which are shown in Figure 3. The diamond projectile reaches the Ti_2_C membrane at ~7.5 ps, and significant kinetic energy is transmitted to the impact region (Figure 3a,b). A maximum out-of-plane deflection of ~33.94 Å is reached at 22.5 ps (Figure 3c). The projectile remains in contact with the nanosheet for a short period and then disengages at ~37.5 ps (Figure 3d). Thereafter, the local deformation propagates to the boundary, and the nanosheet resumes to a flat status with obvious out-of-plane vibration (Figure 3e,f). Similar deformation phenomena are observed from the Ti_3_C_2_ and Ti_4_C_3_ nanosheets, though the maximum out-of-plane deflections are significantly smaller—about ~26.31 Å and ~20.01, respectively.

**Figure 4:**
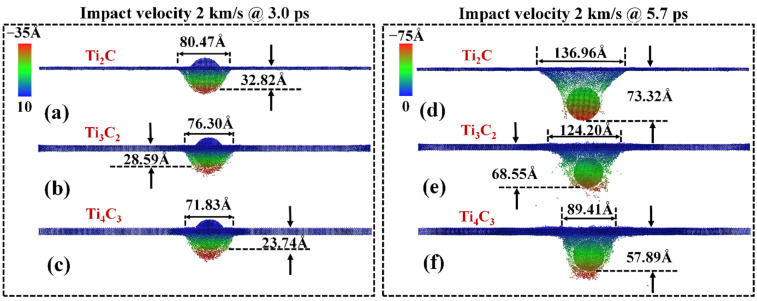
Side view of the z-direction deformation in Ti_n+1_C_n_ under an impact velocity of 2 km/s. Atomic configurations at a simulation time of 3.0 ps: (**a**) Ti_2_C; (**b**) Ti_3_C_2_; (**c**) Ti_4_C_3_. Atomic configuration at a simulation time of 5.7 ps: (**d**) Ti_2_C; (**e**) Ti_3_C_2_; (**f**) Ti_4_C_3_. Atoms are colored according to their coordinates in the thickness direction.

As the impact velocity increases to ~1 km/s, local damage of the nanosheets is observed for all the examined samples, and the projects are trapped in the damaged area. The perforation is observed when the velocity is increased to ~1.2, 1.4 and 1.6 km/s for the Ti_2_C, Ti_3_C_2_ and Ti_4_C_3_ nanosheets, respectively. Figure 4 shows the failure scenario of the nanosheets subject to an impact velocity of 2 km/s. As can be seen, cracks initiate from the impact area with the accumulated stress, and the thinnest Ti_2_C nanosheet experiences the largest out-of-plane deformation prior to the complete failure (~32.82 Å, Figure 4a) compared with the Ti_3_C_2_ and Ti_4_C_3_ counterparts (Figure 4b,c). After perforation at 5.7 ps (Figure 4d–f), more discrete debris is generated from the Ti_3_C_2_ and Ti_4_C_3_ nanosheets. As expected, the thicker nanosheet adsorbs more kinetic energy, and, thus, the projectile exhibits a shorter travel distance at the same simulation time of 5.7 ps.

**Figure 5:**
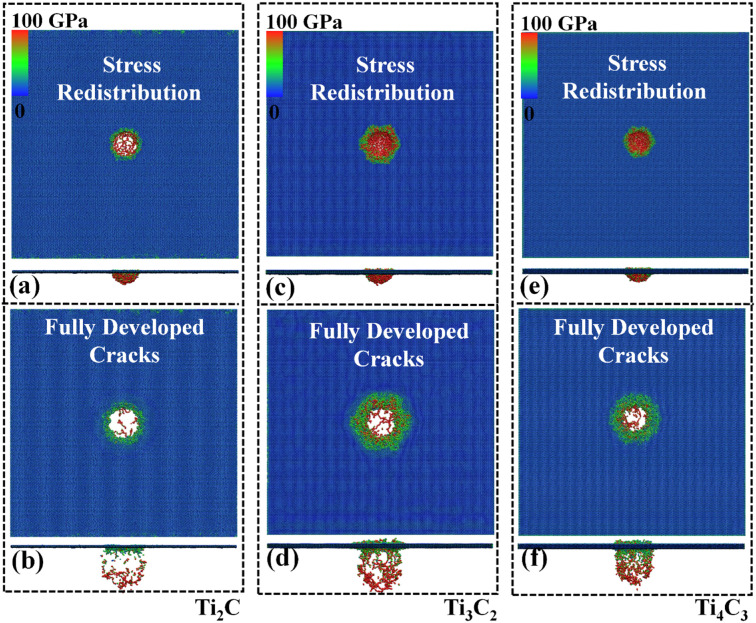
von Mises stress distribution of the MXene nanosheets under an impact velocity of 5 km/s. Upper panels and bottom panels are the top and side views of the nanosheets, respectively. Atomic configuration of Ti_2_C nanosheets at simulation times of: (**a**) 0.7 ps and (**b**) 1.9 ps. Atomic configuration of Ti_3_C_2_ nanosheets at simulation times of: (**c**) 0.7 ps and (**d**) 1.9 ps. Atomic configuration of Ti_4_C_3_ nanosheets at simulation times of: (**e**) 0.7 ps and (**f**) 1.9 ps. For all figures, the upper panel is the top view and the lower panel is the front view.

Severer local deformation is observed when the impact velocity increases further. The amount of discrete debris in nanosheets presents a positive correlation with impact velocity. Under the high impact velocity of ~5 km/s, the contact region melts immediately as the projectile reaches the nanosheet and the high impact energy creates a lot of small discrete debris for all samples. Among all three samples, Ti_4_C_3_ owns the largest number of atoms and, consistently, impact Ti_4_C_3_ leads to the largest amount of discrete debris for an impact velocity above 5 km/s (Figure 5). During the crack propagation phase, the accumulated stress (at the impact region) starts to re-distribute, and the stress distribution in the deformed region of Ti_2_C and Ti_4_C_3_ exhibits a circular pattern (Figure 5b,f), while the Ti_3_C_2_ nanosheet demonstrates a hexagonal pattern (Figure 5d). This phenomenon is considered to be a result of the lattice structure and lattice orientation.

Differently from the armchair/zigzag kicking fracture mechanisms and significant crack propagation phenomena observed in carbonaceous nanosheets [17,38,39], the cracks in Ti_n+1_C_n_ propagate slightly along all directions. The shape and size of the damaged area are generally the same during the short crack expansion period; this phenomenon is related to Ti_n+1_C_n_ MXenes’ metal-like, plastic deformation energy adsorption mechanism. Recall the stress-strain curve of Ti_n+1_C_n_—as the ultimate stress reaches locally, a total failure in MXenes is not observed immediately [40,41]. However, the stress-strain curve of carbonaceous nanosheets usually drops directly to 0 upon ultimate stress, suggesting brittle behavior, which is consistent with their large crack propagation phenomena [42]. Additional simulations conducted by using a time step of 0.1 fs to 0.5 fs yield the same observations.

### 3.2. Stress Distribution and Propogation

**Figure 6:**
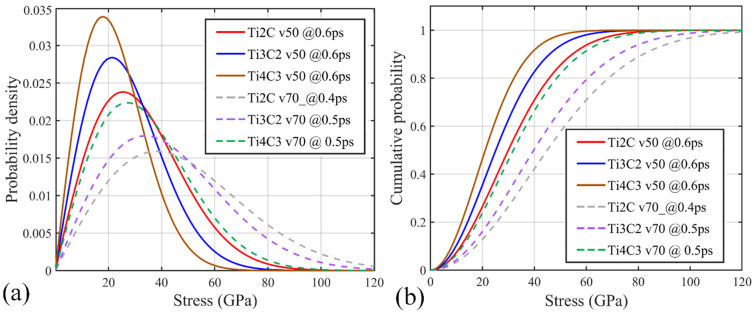
The distribution characteristics of the von Mises stress of Ti_n+1_C_n_ nanosheets under impact prior to bond break. (**a**) The probability density of the von Mises atomic stress; and (**b**) the cumulative density of the von Mises atomic stress.

With the above deformation understanding of Ti_n+1_C_n_ nanosheets under impact, we then analyze the stress distribution and propagation feature within the MXenes. For the same impact velocity amplitude, the probability densities of the von Mises stress (prior to bond break) for the three MXene nanosheets share a similar unimodal distribution pattern (Figure 6a). Subjected to the same impact velocity, the thinnest Ti_2_C nanosheet possesses the lowest probability density peak at a higher stress magnitude, as it experiences larger deformation prior to the bond break compared with the thicker samples.

As the impact velocity increases, the peak of the probability density curve for each sample declines, and its location shifts to a higher stress magnitude (dash lines in Figure 6a). Larger impact velocities result in severer stress concentrations at the impact region. Meanwhile, the melting atoms or the generation of debris adversely influence the stress propagation; therefore, the probability density profile becomes flattened at higher impact velocities. Such observation agrees well with that reported for other nanostructures [10,43].

Theoretically, the elastic stress wave velocity $v_{s}$ in a solid material can be calculated based on its Young’s modulus *E* and density *ρ* according to $v_{s}=\sqrt{E/\rho }$ [44,45]. Alternatively, $v_{s}$ can be estimated by tracking the location of the highest von Mises atomic stress during the simulation [39]. According to the simulation results, the elastic wave propagation velocity along the in-plane X direction is about 10.21, 9.31 and 9.20 km/s for Ti_2_C, Ti_3_C_2_ and Ti_2_C, respectively. Along the in-plane Y direction, it is about 10.81, 9.32 and 9.11 km/s, respectively. The estimated wave velocity agrees well with the theoretical calculation based on the reported Young’s modulus [4,40,41]. The cumulative density of the von Mises atomic stress prior to the bond break presented in Figure 6b also agrees well with the above finding: the curve slopes of the three materials subject to the same impact velocity suggest that Ti_2_C is likely to bear more loading prior to the bond break. It also suggests that Ti_4_C_3_ has the least significant breaking stress among all three materials.

### 3.3. Impact Resistance Evaluation

**Figure 7:**
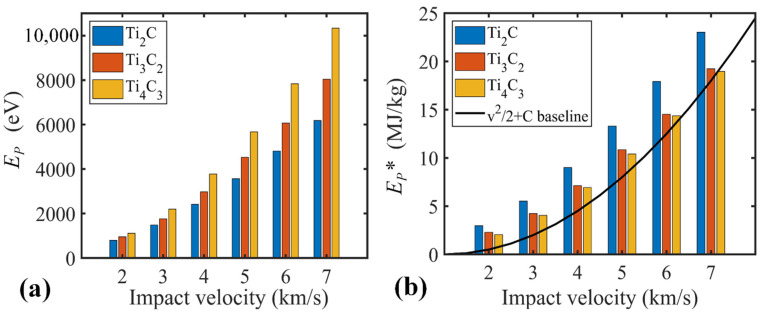
Performance of MXene nanosheets impact with the projectile of various velocity amplitudes. (**a**) Penetration energy and (**b**) specific penetration energy as a function of impact velocity for Ti_2_C, Ti_3_C_2_ and Ti_4_C_3_ nanosheets.

To quantitatively evaluate the impact resistance of Ti_n+1_C_n_ nanosheets, its penetration ($E_{p}$) and specific penetration energy$\;E_{p}^{*}$ under various velocity amplitudes are calculated and compared. According to Figure 7a, the penetration energies of the three 2D titanium carbide MXene nanosheets increase similarly in the examined impact velocity ranging from 2 to 7 km/s. Specifically, the Ti_4_C_3_ nanosheet shows the largest $E_{p}$, suggesting a stronger impact resistance.

**Figure 8:**
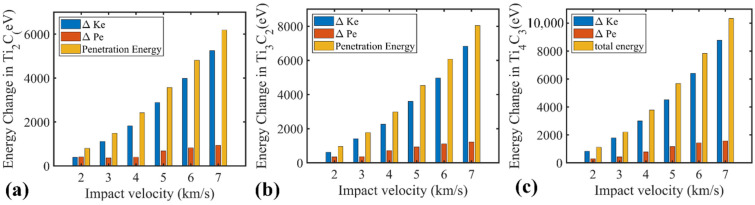
Energy profile of the MXene nanosheet down for (**a**) Ti_2_C; (**b**) Ti_3_C_2_; (**c**) Ti_4_C_3_. The blue, orange and yellow bar represent the kinetic energy gain, potential energy gain and penetration energy, respectively.

For nanosheets with a thickness *h* far less than the projectile diameter *D* (i.e., *D*/*h* >> 1) [20], the nanosheet can be treated as a thin film, and its penetration energy can be estimated from $E_{p}=\left(\rho A_{s}h\right)v^{2}/2+E_{d}$. The first term refers to the minimum inelastic energy or kinetic energy transferred to the target nanosheet (where $A_{s}$ represents the strike face area and v stands for the impact velocity). The second term $E_{d}$ represents other energy dissipation mechanisms, such as elastic deformation and the break of bonds. Further, the specific or gravimetric penetration energy that is defined as$\;E_{p}^{*}=E_{p}/\left(\rho A_{s}h\right)$ can be adopted to evaluate the impact resistance capability of different samples. The gravimetric penetration energy can be further expressed as $E_{P}^{*}=v^{2}/2+E_{d}^{*}$. Apparently, $E_{d}^{*}$ is a figure of merit to evaluate the impact energy delocalization ability, which is dominated by the elastic wave propagation velocity. A stronger energy delocalization ability alleviates the local stress concentration within the nanosheet and thus enhances its impact resistance. As compared in Figure 7b, the estimated $E_{P}^{*}$ of the examined samples generally follows the material-independent energy dissipation baseline (i.e., $v^{2}/2$). Under low impact velocities (<2 km/s), Ti_2_C exhibits higher $E_{P}^{*}$, which is anticipated to be caused by the significant local deformation prior to the bond break (Figure 4). Such observation is affirmed by the energy break down shown in Figure 8, from which the potential energy increase for the Ti_2_C nanosheet is comparable with the kinetic energy. As the impact velocity increases above 3 km/s, the elastic wave propagation velocity or energy delocalization ability become less dominant in determining the impact resistance ability of the nanosheet, due to the severe local deformation in the contact region (Figure 5). Different Ti_n+1_C_n_ MXene nanosheets share a similar Young’s modulus [10,43], while the Ti_2_C nanosheet has the lowest mass. Such fact makes Ti_2_C exhibit a higher specific penetration energy than the other two counterparts, and the advantage becomes more evident under higher impact velocities (Figure 7b).

## 4. Conclusions

In summary, the fracture behaviors of monolayer Ti_n+1_C_n_ MXene nanosheets subject to various impact velocities are explored. For low impact velocities less than 1 km/s, all of the tested samples experience significant deformation, suggesting elastic behavior. After increasing the impact velocity above its penetration threshold, Ti_2_C presents the most significant out-of-plane deformation prior to perforation, followed by Ti_3_C_2_ and Ti_4_C_3_ which is consistent with the ranking of elastic wave propagation velocities. Elastic wave propagation velocity is an important figure in impact resistance assessment, as it positively correlates with the energy delocalization rate, which helps materials to deform more and thus absorb more energy prior to the bond break. As can be seen by tracking the atomic Von Mises stress distribution, Ti_2_C possesses the most significant elastic wave propagation velocities, which are calculated to be 10.21 and 10.81 km/s for the X and Y direction, respectively. Though Ti_4_C_3_ shows an advantage in $E_{p}$ among the test samples, Ti_2_C nanosheets present superior $E_{P}^{*}$ for all the tested velocity amplitudes, which indicates a negative correlation between $E_{P}^{*}$ and the number *n* in the formula Ti_n+1_C_n_. This study provides a fundamental understanding of the deformation and penetration mechanisms of Ti_n+1_C_n_ nanosheets under impact, which should shed light on the design of MXenes-related composites for bullet-proof application or shielding structures for aerospace systems protection applications for Ti_n+1_C_n_-related composites. These results of Ti_n+1_C_n_ MXene nanosheets were obtained under a temperature of 10K or failure mechanisms under a higher temperature. Large-scale monolithic Ti_n+1_C_n_ MXene nanosheets still deserve further investigation.

## Data Availability

The data presented in this study are available in this article.
